# “Beenomials”: exploring beekeeping as a rehabilitation tool in the field of mental health

**DOI:** 10.1192/j.eurpsy.2024.319

**Published:** 2024-08-27

**Authors:** A. Barbieri

**Affiliations:** Department of Mental Health, ASL CN1, Cuneo, Italy

## Abstract

**Introduction:**

Beekeeping is a peculiar activity able to connect people both to nature and to other people. Extant research shows how it provides beekeepers with meaning, opportunities for learning, and a sense of connection to bees as well as to the surrounding ecosystem. The relationship of care and interdependence that is established supports well-being, encourages collaboration and positive social relations.

**Objectives:**

“Beenomies” is a pilot project inspired by the union of opposites symbolically associated with bees: love and war, sweetness (honey) and bitterness (venom), the individual and multiplicity (society), regeneration and death. As CG Jung observed, honey expresses, psychologically, “the joy of life and the life urge which overcome […] the dark and the inhibiting. Where spring-like joy and expectation reign, spirit can embrace nature and nature, spirit”. Drawing on this psychological and philosophical basis, the project aimed to introduce beekeeping in a therapeutic community placed in the Alpine environment (Mondovì, Italy), to explore its rehabilitative potential and its ability to promote well-being in the field of mental health.

**Methods:**

The project stems from the collaboration between mental health services, a local agriculture high school, and a farm involved in social agriculture. Initially, some beehives have been settled on the land surrounding the therapeutic community. Activities of beekeeping have been conducted and supervised by experienced beekeepers of the farm involved, who engaged a selected group of users hosted in the community (n=15), instructed them and worked side by side for several weeks, according to the bees’ needs and the seasonal rhytms. Once the training was completed, teaching activities have been co-conducted by beekeepers and participants, to introduce and train a group of students from the local agriculture high school. A study encompassing observational data, surveys, and semi-structured interviews was conducted to monitor and evaluate the project as it unfolded.

**Results:**

The performance of practical activities (i.e. beekeeping operations) proved successful in relaxing social norms around talking, lowering the emotional intensity of the encounter, allowing non-verbal communication and normalizing silence. These features supported participants with relational difficulties and encouraged the gradual development of skills in the social area. In the second part of the project, the involvement of high school students that needed to be trained allowed participants to have an active role as teachers; this contributed to the development of positive feelings, increased self-esteem and self-efficacy, eventually supporting the recovery process.

**Conclusions:**

Preliminary findings suggest further collaboration between different social actors and further research to develop inclusive, effective, and community-based interventions in the field of mental health and rehabilitation.

**Disclosure of Interest:**

None Declared

